# Supplementary material for: Clinical Application of Comprehensive Genomic Profiling Tests for Diffuse Gliomas
Source: Cancers (Basel). 2022 May 16;14(10):2454. doi: 10.3390/cancers14102454 (PMC9139713; doi:10.3390/cancers14102454)
Supplement: Supplementary file 1 [file cancers-14-02454-s001.zip › cancers-1674301-SI.pdf]

cancers

MDPI

Supplementary Materials: Clinical Application of Comprehensive Genomic Profiling Tests for Diffuse Gliomas

Takaki Omura, Masamichi Takahashi, Makoto Ohno, Yasuji Miyakita, Shunsuke Yanagisawa, Yukie Tamura, Miyu Kikuchi, Daisuke Kawauchi, Tomoyuki Nakano, Tomohiro Hosoya, Hiroshi Igaki, Kaishi Satomi, Akihiko Yoshida, Kuniko Sunami, Makoto Hirata, Tatsunori Shimoi, Kazuki Sudo, Hitomi S. Okuma, Kan Yonemori, Hiromichi Suzuki, Koichi Ichimura and Yoshitaka Narita *

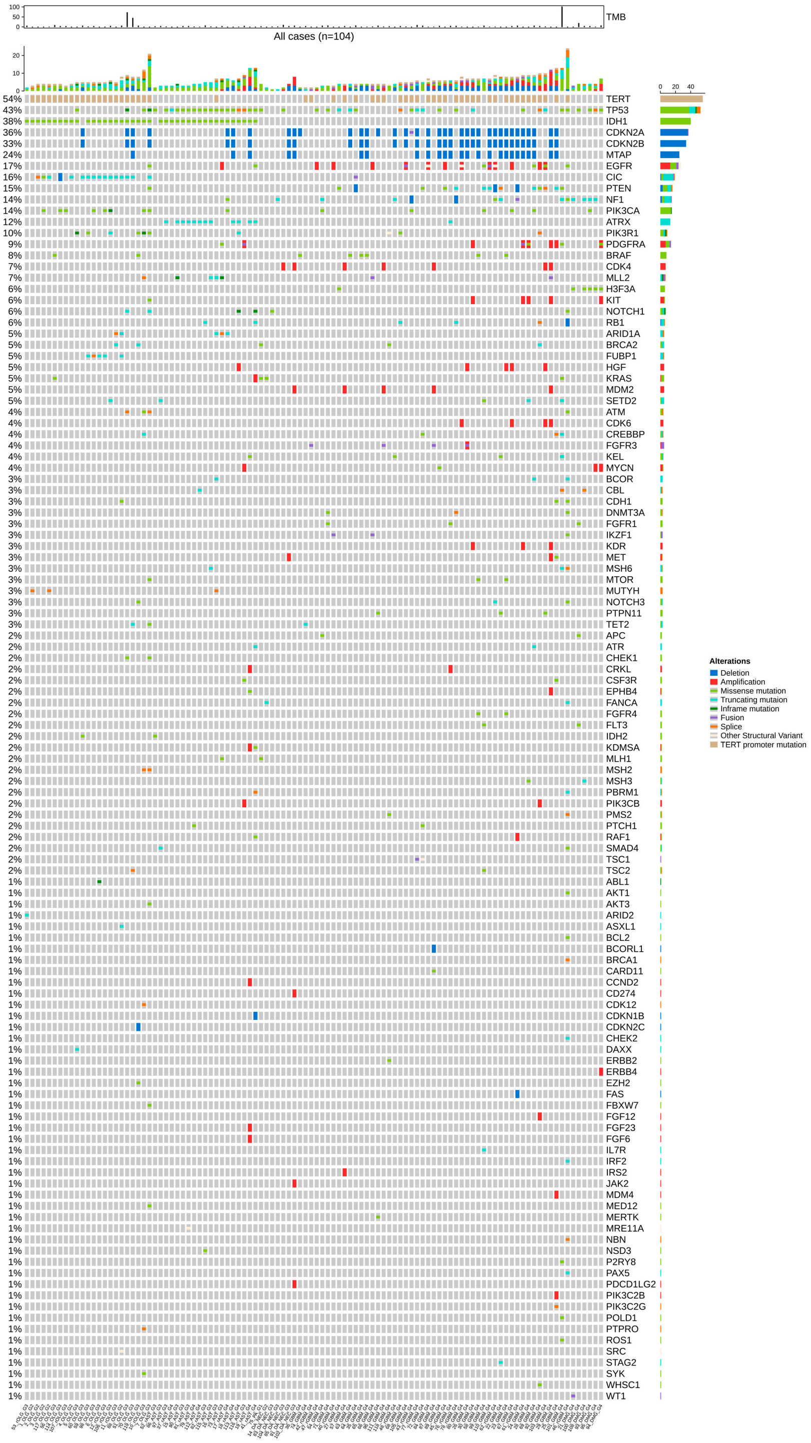

(A)

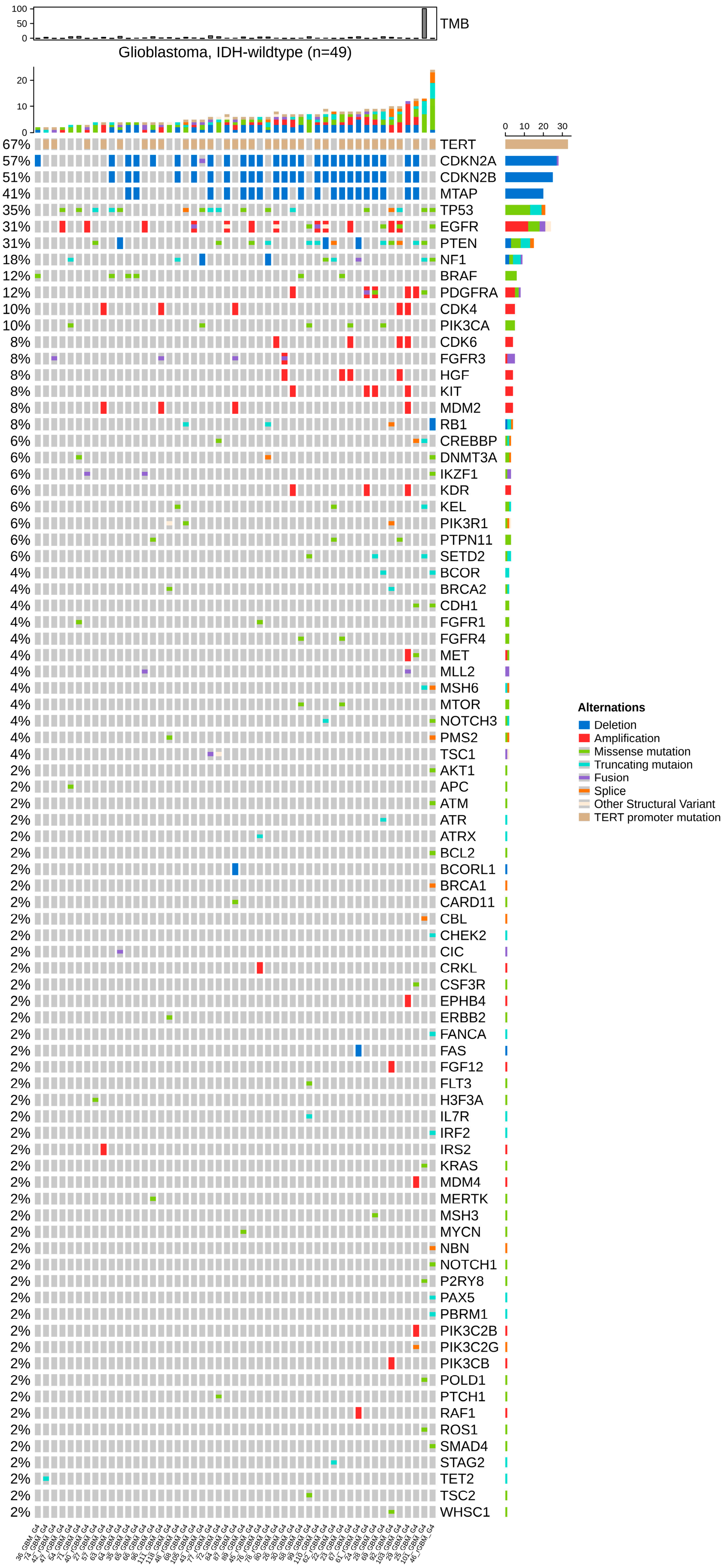

TMB
Glioblastoma, IDH-wildtype (n=49)
67% TERT
57% CDKN2A
51% CDKN2B
41% MTAP
35% TP53
31% EGFR
31% PTEN
18% NF1
12% BRAF
12% PDGFRA
10% CDK4
10% PIK3CA
8% CDK6
8% FGFR3
8% HGF
8% KIT
8% MDM2
8% RB1
6% CREBBP
6% DNMT3A
6% IKZF1
6% KDR
6% KEL
6% PIK3R1
6% PTPN11
6% SETD2
4% BCOR
4% BRCA2
4% CDH1
4% FGFR1
4% FGFR4
4% MET
4% MLL2
4% MSH6
4% MTOR
4% NOTCH3
4% PMS2
4% TSC1
2% AKT1
2% APC
2% ATM
2% ATR
2% ATRX
2% BCL2
2% BCORL1
2% BRCA1
2% CARD11
2% CBL
2% CHEK2
2% CIC
2% CRKL
2% CSF3R
2% EPHB4
2% ERBB2
2% FANCA
2% FAS
2% FGF12
2% FLT3
2% H3F3A
2% IL7R
2% IRF2
2% IRS2
2% KRAS
2% MDM4
2% MERTK
2% MSH3
2% MYCN
2% NBN
2% NOTCH1
2% P2RY8
2% PAX5
2% PBRM1
2% PIK3C2B
2% PIK3C2G
2% PIK3CB
2% POLD1
2% PTCH1
2% RAF1
2% ROS1
2% SMAD4
2% STAG2
2% TET2
2% TSC2
2% WHSC1
Alternations
Deletion
Amplification
Missense mutation
Truncating mutaion
Fusion
Splice
Other Structural Variant
TERT promoter mutation

(B)

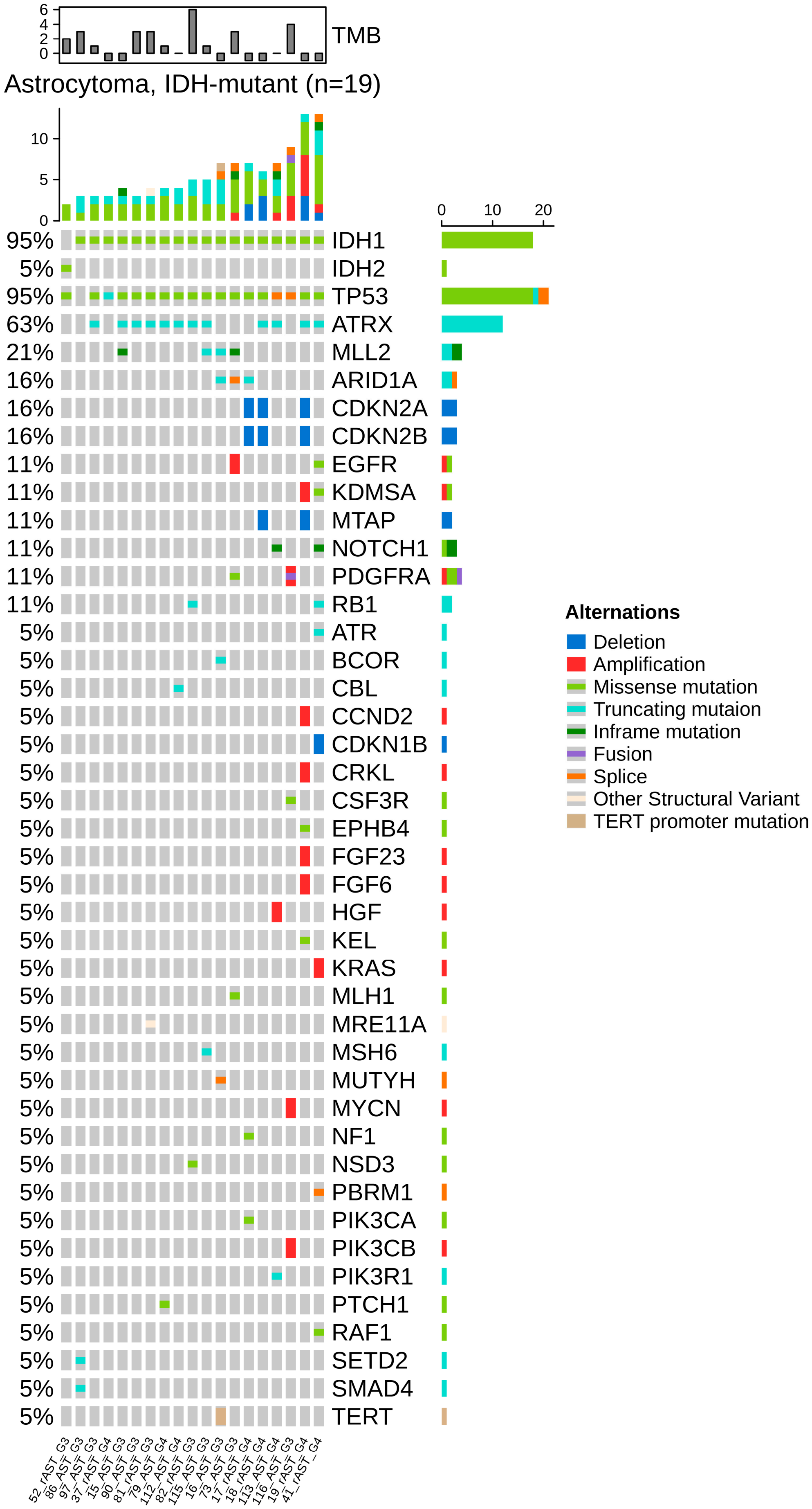
TMB
0
2
4
6
Astrocytoma, IDH-mutant (n=19)
0
5
10
0
10
20
95% IDH1
5% IDH2
95% TP53
63% ATRX
21% MLL2
16% ARID1A
16% CDKN2A
16% CDKN2B
11% EGFR
11% KDMSA
11% MTAP
11% NOTCH1
11% PDGFRA
11% RB1
5% ATR
5% BCOR
5% CBL
5% CCND2
5% CDKN1B
5% CRKL
5% CSF3R
5% EPHB4
5% FGF23
5% FGF6
5% HGF
5% KEL
5% KRAS
5% MLH1
5% MRE11A
5% MSH6
5% MUTYH
5% MYCN
5% NF1
5% NSD3
5% PBRM1
5% PIK3CA
5% PIK3CB
5% PIK3R1
5% PTCH1
5% RAF1
5% SETD2
5% SMAD4
5% TERT
52_rAST_G3
86_AST_G3
97_AST_G3
37_rAST_G4
15_AST_G3
90_AST_G3
81_rAST_G3
79_AST_G4
112_AST_G4
82_rAST_G3
115_AST_G3
16_AST_G3
73_AST_G3
17_rAST_G4
18_rAST_G4
113_AST_G4
116_AST_G3
19_rAST_G4
41_rAST_G4
Alternations
Deletion
Amplification
Missense mutation
Truncating mutaion
Inframe mutation
Fusion
Splice
Other Structural Variant
TERT promoter mutation

(C)

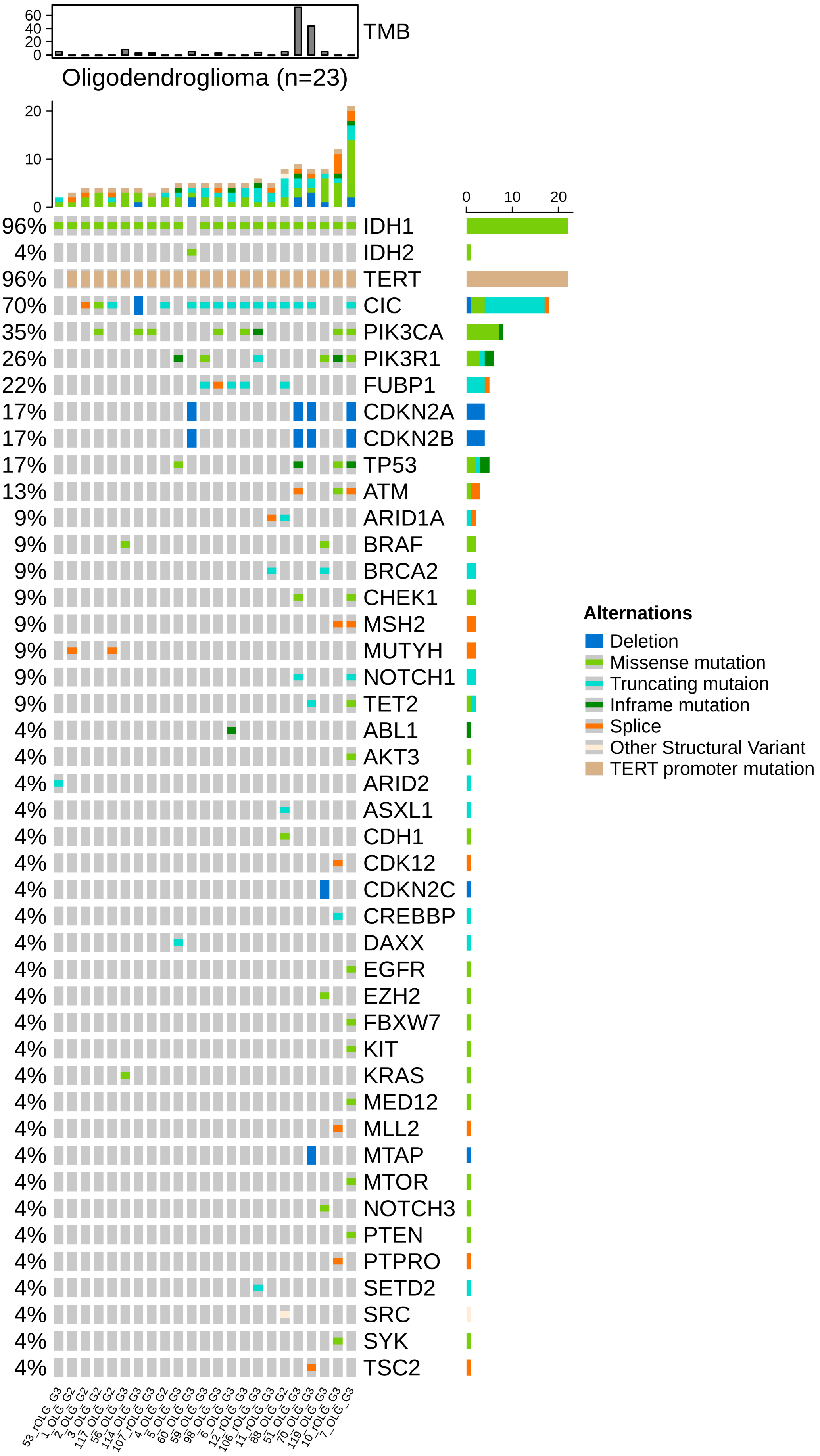

TMB
Oligodendroglioma (n=23)
96% IDH1
4% IDH2
96% TERT
70% CIC
35% PIK3CA
26% PIK3R1
22% FUBP1
17% CDKN2A
17% CDKN2B
17% TP53
13% ATM
9% ARID1A
9% BRAF
9% BRCA2
9% CHEK1
9% MSH2
9% MUTYH
9% NOTCH1
9% TET2
4% ABL1
4% AKT3
4% ARID2
4% ASXL1
4% CDH1
4% CDK12
4% CDKN2C
4% CREBBP
4% DAXX
4% EGFR
4% EZH2
4% FBXW7
4% KIT
4% KRAS
4% MED12
4% MLL2
4% MTAP
4% MTOR
4% NOTCH3
4% PTEN
4% PTPRO
4% SETD2
4% SRC
4% SYK
4% TSC2
Alternations
Deletion
Missense mutation
Truncating mutaion
Inframe mutation
Splice
Other Structural Variant
TERT promoter mutation

(D)

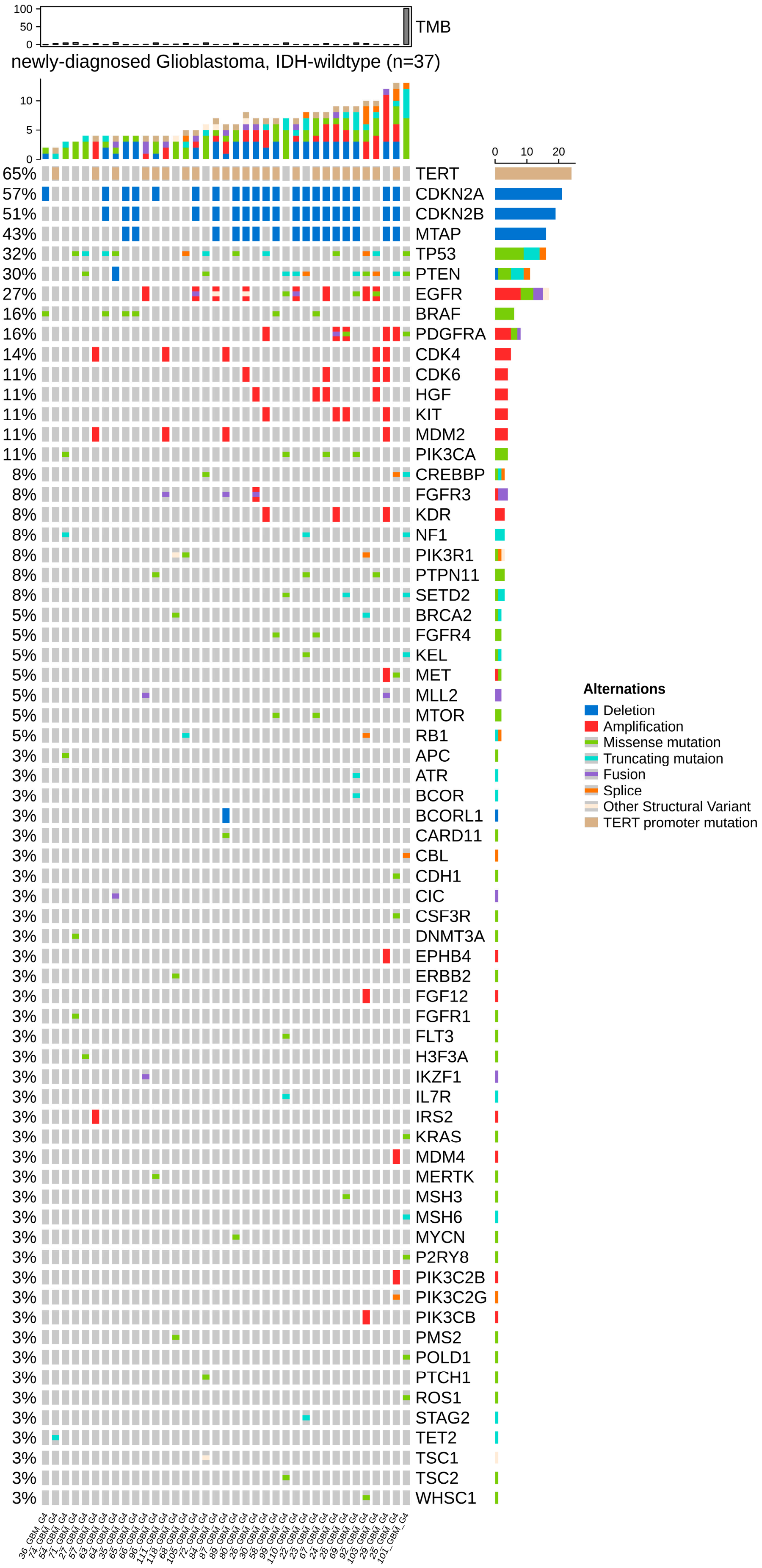
TMB
newly-diagnosed Glioblastoma, IDH-wildtype (n=37)
65% TERT
57% CDKN2A
51% CDKN2B
43% MTAP
32% TP53
30% PTEN
27% EGFR
16% BRAF
16% PDGFRA
14% CDK4
11% CDK6
11% HGF
11% KIT
11% MDM2
11% PIK3CA
8% CREBBP
8% FGFR3
8% KDR
8% NF1
8% PIK3R1
8% PTPN11
8% SETD2
5% BRCA2
5% FGFR4
5% KEL
5% MET
5% MLL2
5% MTOR
5% RB1
3% APC
3% ATR
3% BCOR
3% BCORL1
3% CARD11
3% CBL
3% CDH1
3% CIC
3% CSF3R
3% DNMT3A
3% EPHB4
3% ERBB2
3% FGF12
3% FGFR1
3% FLT3
3% H3F3A
3% IKZF1
3% IL7R
3% IRS2
3% KRAS
3% MDM4
3% MERTK
3% MSH3
3% MSH6
3% MYCN
3% P2RY8
3% PIK3C2B
3% PIK3C2G
3% PIK3CB
3% PMS2
3% POLD1
3% PTCH1
3% ROS1
3% STAG2
3% TET2
3% TSC1
3% TSC2
3% WHSC1
Alternations
Deletion
Amplification
Missense mutation
Truncating mutaion
Fusion
Splice
Other Structural Variant
TERT promoter mutation

(E)

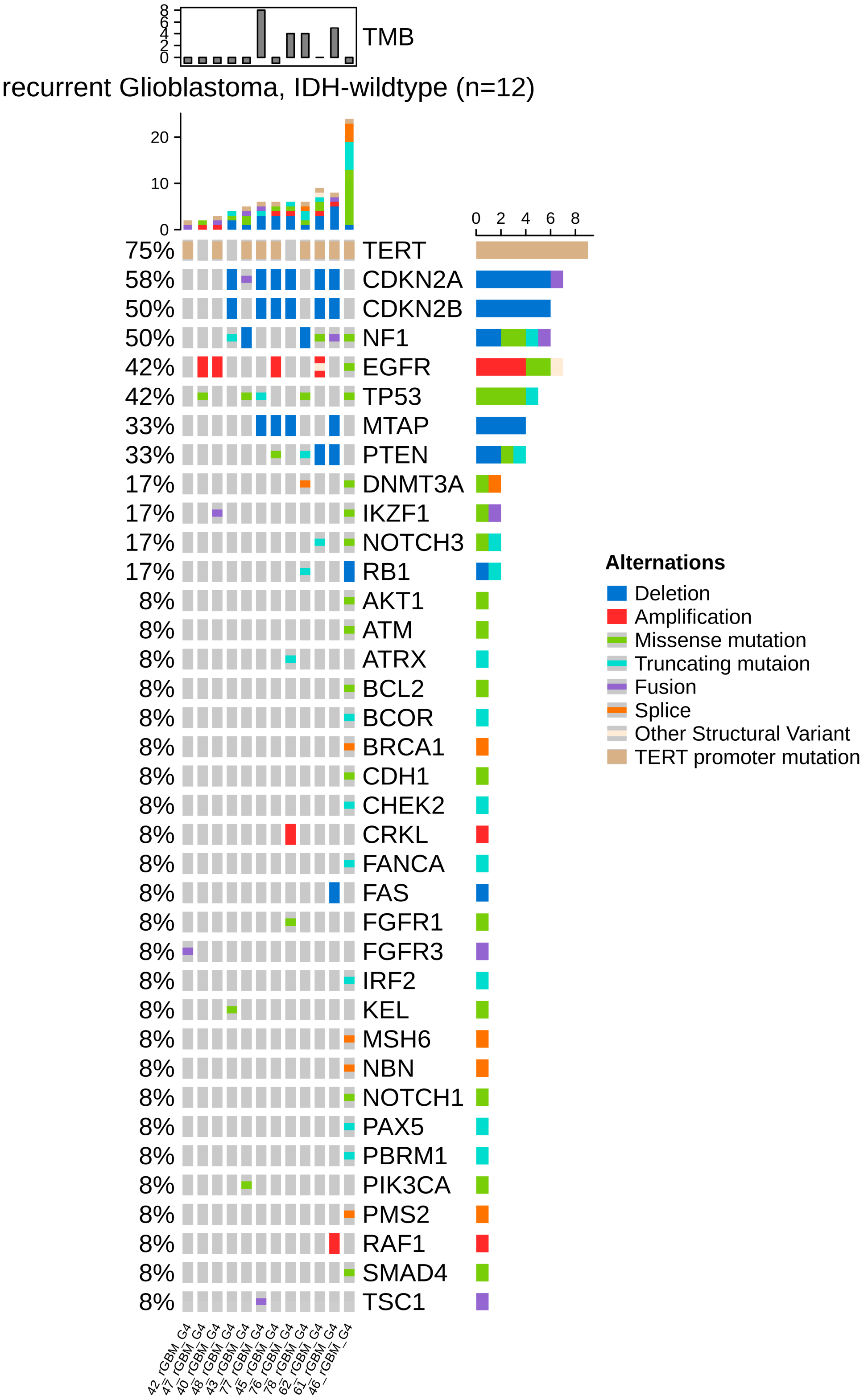

(F)

Figure S1. Full version of oncoprint for (**A**) all the 104 patients in this cohort and patients with (**B**) *IDH*-wt glioblastoma, (**C**) *IDH*-mt astrocytoma, (**D**) Oligodendroglioma, *IDH*-mt, 1p19q co-deletion, (**E**) newly diagnosed *IDH*-wt glioblastoma, and (**F**) recurrent *IDH*-wt glioblastoma.

Table S1. Genomic profiling tests available in Japan.

	**OncoGuide NCC OncoPanel System**	**FoundationOne CDx Cancer Genomic Profile**
Specimens	Tumor (FFPE) + peripheral blood	Tumor (FFPE)
Sequencing	DNA only	DNA only
Tumor purity	at least ≥20%	≥30% recommended
Number of genes	114	324
Aberrations	mutation, CNA, fusion, SV	mutation, CNA, fusion, SV
Companion diagnosis	no	Yes

CNA: copy number alteration, SV: Structural Variant.

Table S2. Clinical actionability in the 65 patients with glioma, including *IDH*-mt astrocytoma and oligodendroglioma *IDH*-mt, 1p19q co-deletion.

**No.**	**ID (in this Cohort)**	**CGPT**	**Diagnosis**	**Age**	**Gender**	**Acctionable Gene Aberration**	**Therapeutic Agents**	**Drug Type**
1	51_OLG	F-One	Oligodendroglioma, *IDH*-mt, G3	52	F	*ATM* splice site 6573-1G>A	ATR inhibitor	Investigational drug
2	58_GBM	F-One	GBM, *IDH*-wt	40	M	*BRAF* V600E	Dabrafenib/Trametinib	clinical trial
3	63_GBM	F-One	GBM, *IDH*-wt	62	F	*BRAF* V600E	Dabrafenib/Trametinib	clinical trial
4	65_GBM	F-One	GBM, *IDH*-wt	52	F	*BRAF* V600E	Dabrafenib/Trametinib	clinical trial
5	90_AST	F-One	Astrocytoma, *IDH*-mt, G3	41	M	*IDH1* R132H	*IDH1* inhibitor	Investigational drug
6	71_GBM	F-One	GBM, *IDH*-wt	55	F	*FGFR1* K656E	*FGFR* inhibitor	Investigational drug *
7	87_GBM	F-One	GBM, *IDH*-wt	59	M	*FGFR3 FGFR3-TACC3* fusion	*FGFR* inhibitor	Investigational drug *
8	100_DMG	F-One	DMG	33	M	*FGFR1* N546K, TMB high	*FGFR* inhibitor, Pembrolizumab	Investigational drug *, clinical trial *
9	101_GBM	F-One	GBM, *IDH*-wt	59	F	*MSH6* C694fs*4, *MSH6* I795fs*15	Pembrolizumab	clinical trial *
10	111_GBM	F-One	GBM, *IDH*-wt	53	M	*FGFR3 FGFR3-TACC3* fusion	*FGFR* inhibitor	Investigational drug *
11	117_OLG	F-One	Oligodendroglioma, *IDH*-mt, G2	34	M	*IDH1* R132H	*IDH1* inhibitor	Investigational drug *
12	119_OLG	F-One	Oligodendroglioma, *IDH*-mt, G3	52	F	BRAF G469R, BRCA2 I682fs*48	small molecule drug, Pembrolizumab/Olaparib	Investigational drug *, clinical trial *

DMG: diffuse midline glioma, (*): enrollment in a clinical trial is pending.
